# Nuclear Receptor Interacting Protein-2 Mediates the Stabilization and Activation of β-Catenin During Podocyte Injury

**DOI:** 10.3389/fcell.2021.781792

**Published:** 2021-12-24

**Authors:** Qing Hou, Weibo Le, Shuyan Kan, Jinsong Shi, Yue Lang, Zhihong Liu, Zhaohong Chen

**Affiliations:** ^1^ National Clinical Research Center of Kidney Disease, Jinling Clinical College, Southeast University School of Medicine, Nanjing, China; ^2^ National Clinical Research Center of Kidney Disease, Jinling Hospital, Nanjing University School of Medicine, Nanjing, China

**Keywords:** podocyte injury, proteinuria, NRIP2, β-catenin, degradation

## Abstract

**Objective:** Activation of β-catenin causes podocyte injury and proteinuria, but how β-catenin signalling is regulated during podocyte injury remains elusive. Nuclear receptor interacting protein 2 (NRIP2) modulates the Wnt pathway in colorectal cancer-initiating cells, but the role of NRIP2 in podocyte injury has not yet been investigated. We aimed to examine the interaction between NRIP2 and β-catenin signalling.

**Materials and Methods:** Knockdown or overexpression of NRIP2 and β-catenin and chemical treatments were performed in cultured human podocytes. Immunoprecipitation, immunoblotting and immunofluorescence assays were used to assess protein interactions and expression. Data from the GEO dataset and kidney tissues from patients with focal segmental glomerulosclerosis (FSGS) and surgical nephrectomy were examined. An adriamycin (ADR) nephropathy model was established in NRIP2 knockout mice.

**Results:** NRIP2 knockdown accelerated β-catenin degradation, which was reversed by MG132; specifically, NRIP2 bound β-catenin and stabilized it to prevent its degradation through the ubiquitin proteasomal pathway. Overexpression of NRIP2 led to β-catenin activation and Snail1 induction, and these effects were attenuated by β-catenin knockdown. NRIP2 knockdown blocked ADR-stimulated β-catenin activation. In ADR mice, genetic knockout of Nrip2 ameliorated podocyte injury and loss, glomerulosclerosis, and proteinuria by inhibiting β-catenin activation. Moreover, NRIP2 was significantly upregulated in podocytes of FSGS patients and colocalized with nuclear β-catenin.

**Conclusion:** These results established NRIP2 as a stabilizer of β-catenin activation through the ubiquitin proteasomal pathway in podocyte injury.

## Introduction

Podocyte injury has been highlighted as the central cellular event in proteinuria development in nephrotic syndrome (NS) (Saleem, 2019; Saleem and Welsh, 2019; Kopp et al., 2020). Constitutive activation of β-catenin signalling strongly promotes the progression of podocyte injury, proteinuria and glomerulosclerosis (Dai et al., 2009; Heikkilä et al., 2010; Zhou and Liu, 2015; Bose et al., 2017). β-Catenin, a master transcriptional modulator, is induced and activated in podocytes from patients with various proteinuric kidney diseases. β-Catenin controls the expression of several key mediators implicated in podocytopathies, such as the renin–angiotensin system (Zhou et al., 2015a), Snail1 (Wang et al., 2011) and multiple mesenchymal markers (He et al., 2010). Importantly, overexpression of β-catenin *in vitro* induces WT1 protein degradation through the ubiquitin–proteasome pathway, and this degradation can be blocked by MG132 (Zhou et al., 2015b). Existing evidence shows that targeting β-catenin signalling with Klotho (Zhou et al., 2019), DKK1 (Kato et al., 2011; Shkreli et al., 2011) or VDR agonists (Shkreli et al., 2011) and genetic inhibition of β-catenin (Dai et al., 2009) can reduce proteinuria in adriamycin (ADR) nephropathy models.

In the absence of Wnt stimulation, cellular β-catenin expression is limited due to phosphorylation-triggered proteasomal degradation. Phosphorylated β-catenin is recognized by E3 ubiquitin ligases and is thus degraded by the proteasome (Valenta et al., 2012). Upon Wnt stimulation, β-catenin phosphorylation is inhibited to ensure β-catenin stabilization and subsequent nuclear translocation and activation (MacDonald et al., 2009).

Nuclear receptor interacting protein 2 (NRIP2) was initially described as a suppressor of the nuclear receptor (NR) by selectively binding the retinoic acid receptor (RAR) and the thyroid hormone receptor (THR) in a ligand-dependent manner in the mouse brain (Greiner et al., 2000). Recently, NRIP2 was shown to regulate colorectal cancer-initiating cell renewal as a Wnt pathway interactor, functioning as a novel molecule that cooperates with RORβ and HMG box-containing protein 1 (HBP1) to modulate Wnt activity (Wen et al., 2017). To date, the role of NRIP2 in podocyte injury has not been investigated. In the present work, we demonstrated that NRIP2 physically interacts with β-catenin and stabilizes it to prevent its degradation by the proteasome in podocytes. During podocyte injury, upregulated NRIP2 binds to, stabilizes and promotes the activation of β-catenin, leading to podocyte injury. Loss of NRIP2 ameliorates ADR-induced podocyte injury *in vitro* and *in vivo*.

## Materials and Methods

### Antibodies

Rabbit anti-NRIP2 (Affinity, AF0597; IF/IHC: 1:100; WB: 1: 500); Mouse anti-WT1 (Dako, ISO5530; IF: ready-to-use); Rabbit anti-WT1 (Abcam, ab15249; IF: 1:200); Mouse anti-Total β-catenin (BD, 610154; IF/IHC: 1:100); Rabbit anti-Active β-catenin (CST, 8814; WB: 1: 1000); Rabbit anti-Ubiquitin (Proteintech, 10201-2-AP; WB: 1: 500); Rabbit anti-Col1a1 (CST, 72026; WB: 1:1000); Rabbit anti-α-SMA (GeneTex, GTX100034; IF: 1:200; WB: 1: 500); Goat anti-Synaptopodin (Santa Cruz, sc-21537; IF: 1: 300); Rabbit anti-GAPDH (EnoGene, E12-052; WB: 1: 5000); Mouse anti-β-Tubulin (Transgen, HC101-01; WB: 1: 5000).

### siRNA and Plasmid

Negative control, human NRIP2 siRNAs (#1: GUG​UAG​TGT​GAT​UUA​AAG​A; #2: GAU​AGA​GAT​TUU​AGU​TCT​T), human β-catenin siRNA (GGA​UGU​UCA​CAA​CCG​AAU​UTT) were purchased from RIBOBIO Company (Guangzhou, China). NRIP2 and β-catenin (Full length and mutants) were synthesised from GenScript company and cloned into pcDNA3.1 backbone. pRK5-HA-ubiquitin was obtained from MiaoLing Plasmid Sharing Platform (P1761).

### Human Podocyte Culture, Transfection and Immunoblot Analysis

The human podocyte cell line was provided by M. Saleem (Saleem et al., 2002) (University of Bristol, United Kingdom) and was cultured as described previously (Wu et al., 2014). Human podocytes in 6-well plates were ready for transfection. For transfection of plasmids or siRNA, jetPRIME *in vitro* DNA & siRNA transfection reagent (PolyPlus Transfection, pt-114-15) were used according to the manufacturer’s instructions. In brief, 2 µg plasmid was diluted into 200 µl jetPRIME buffer, mixed by vortex. After adding 4 µl jetPRIME reagent and incubating for 10 mins, the transfection mixture was added to the cells in serum containing medium. For siRNA transfection, 110 pmole siRNA was diluted into 200 µl jetPRIME buffer, mixed by vortex. After adding 3 µl jetPRIME reagent and incubating for 10 mins, the transfection mixture was added to the cells in serum containing medium. Cells were harvest at 24 h after transfection. Cells were treated with 10 µM Adriamycin (MCE, HY-15142) for 6 h and 5 µM MG132 (MCE, HY-13259) for 1 h before harvested.

### Immunoprecipitation and Immunoblot Analysis

Human podocytes were lysed in M-PER™ Mammalian Protein Extraction Reagent (Thermo Fisher, 78501) with protease inhibitors, cell lysates were fractionated by SDS-PAGE (Future Biotech, F11412Gel & F15412Gel) and then transferred to polyvinylidene difluoride (PVDF) membranes (Millipore-Merck, United States) for immunoblot analysis.

For Immunoprecipitation, cells were lysed with Minute™ Total Protein Extraction Kit (Invent, SN-002), lysates were incubated with β-catenin antibody with protease inhibitors on a rotator overnight at 4°C. The protein/β-catenin complexes were prepared by adding 15 µl protein A/G Magnetic Beads (MCE, HY-K0202) for 3 h at 4°C. After several washes with cold PBS, the complexes were resuspended with 2 × SDS buffer and boiled for 10 min; supernatants were subjected to SDS-PAGE and immunoblot analysis.

For ubiquitination assays, transfected podocytes treated with or without MG132 were lysed in SN-002 with protease inhibitors, lysates were incubated with primary antibody as indicated on a rotator overnight at 4°C, and subjected to 15 µl protein A/G Magnetic Beads incubation (MCE, HY-K0202) for 3 h at 4°C. After several washes with cold PBS, the complexes were resuspended with 2 × SDS buffer and boiled for 10 min; supernatants were subjected to SDS-PAGE and immunoblot analysis with anti-Ubiquitin antibody.

### Generation of Mice and Measurement of Urine Albumin and Creatinine

Nrip2 Knockout (KO) mice were customized in Cyagen Biosciences Inc. (Suzhou, China). KO mice were generated using CRISPR/Cas9 system (Target-1: TGG​AGC​TAG​TCA​TGC​ACC​TCT​GG and Target-2: AGG​TGG​ACT​AGA​CCG​GAG​AGG​GG) on C57BL/6N background. ADR (Sigma, United States, D1515) were injected at 20 mg/kg body via the tail vein in WT and Nrip2 KO mice at the age of 8-weeks-old, saline-injected mice were served as normal controls. ADR mice were sacrificed at 4 weeks after injection. Urine albumin was quantified by Albuwell M ELISA kit (Exocell, United States, 1011), Urine creatinine were examined with the same samples using Creatinine Companion (Exocell, United States, 1012). The urine albumin excretion was assessed using the ratio of albumin to creatinine. Genotyping primers used were: F1- CAA​AGA​AAG​GAA​GCC​AAG​CTG​GTA​C, R1- ACC​CTG​AGC​CTT​CTA​CCT​GTC​CTA​G, and R2- CTG​CTG​GCT​CCA​TCC​CAA​AAT​AG.

### Kidney Histology

Kidneys of mice were fixed with 4% PFA overnight at 4°C and embedded with paraffin. Paraffin-embedded kidney tissues were cut into 2-μm. Sections were stained with Periodic Acid Schiff (PAS) for histology analysis. Quantification of glomerulosclerosis was measured using ImageJ (NIH).

### Transmission Electron Microscopy

Kidney tissues were fixed in 4% glutaraldehyde with 0.1M cacodylate buffer (pH 7.2) for 4 h. Samples were further rinsed in cacodylate buffer and post-fixed in 1% OsO_4_ with 0.1M cacodylate buffer (pH 7.4) for 24 h, dehydrated in a series of ethanol and infiltrated overnight in a 1:1 mixture of epoxy resin and propylene oxide. Specimens were embedded with Eponate 12 (Ted Pella, United States) and polymerized at 60°C for 24 h. Ultrathin sections were stained with lead citrate and uranyl acetate and viewed on a HITACHI H-7500 microscope.

### Patients’ Enrollment

Patients with FSGS were renal biopsy proven at National Clinical Research Center of Kidney Diseases, Jinling Hospital, Nanjing, China. The clinical characteristics of patients were detailed in Supplementary Table S1. Healthy control glomerular samples were obtained from surgical nephrectomies.

### Immunohistochemistry and Immunofluorescence Staining

Paraffin-embedded kidney sections from human and mouse were deparaffinized, blocked with 10% BSA in PBS for 30 min at room temperature (RT) and then incubated with primary antibodies at 4°C overnight. The next day, after five times washes with PBS, incubate with secondary antibodies at RT for 1 h. For IHC staining, diaminobenzidine (DAB) color reaction was kept with a fixed exposure time for all experiments among the groups. Quantitation of IHC staining in the glomeruli was measured using ImageJ (NIH). For IF staining, Goat anti-Rabbit IgG (H+L) Highly Cross-Adsorbed secondary antibody, Alexa Fluor 488 (ThermoFisher, A-11034, 1:200) and Goat anti-Mouse IgG (H+L) Highly Cross-Adsorbed secondary antibody, Alexa Fluor Plus 555 (ThermoFisher, A32727, 1:200) were incubated for 2 h in dark, and mounted with DAPI- Aqueous, Fluoroshield (Abcam, ab104139) for imaging.

### Statistics

Data were represented as mean ± SD. Data between 2 groups were analysed using with two-tailed unpaired Student’s t test. All experiments were repeated for 3 times, representative experiments were exhibited. *p* < 0.05 were considered statistically significant.

### Study Approval

All protocols regarding the use of human samples were approved by the Human Subjects Committee of Jinling Hospital and written/signed consent was obtained from all patients (2013KLY-013-02). Mice were grown and maintained following standard procedure approved by the Institutional Animal Care and Use Committee at Jinling Hospital.

## Results

### NRIP2 Binds to β-Catenin in Human Podocytes

**FIGURE 1:**
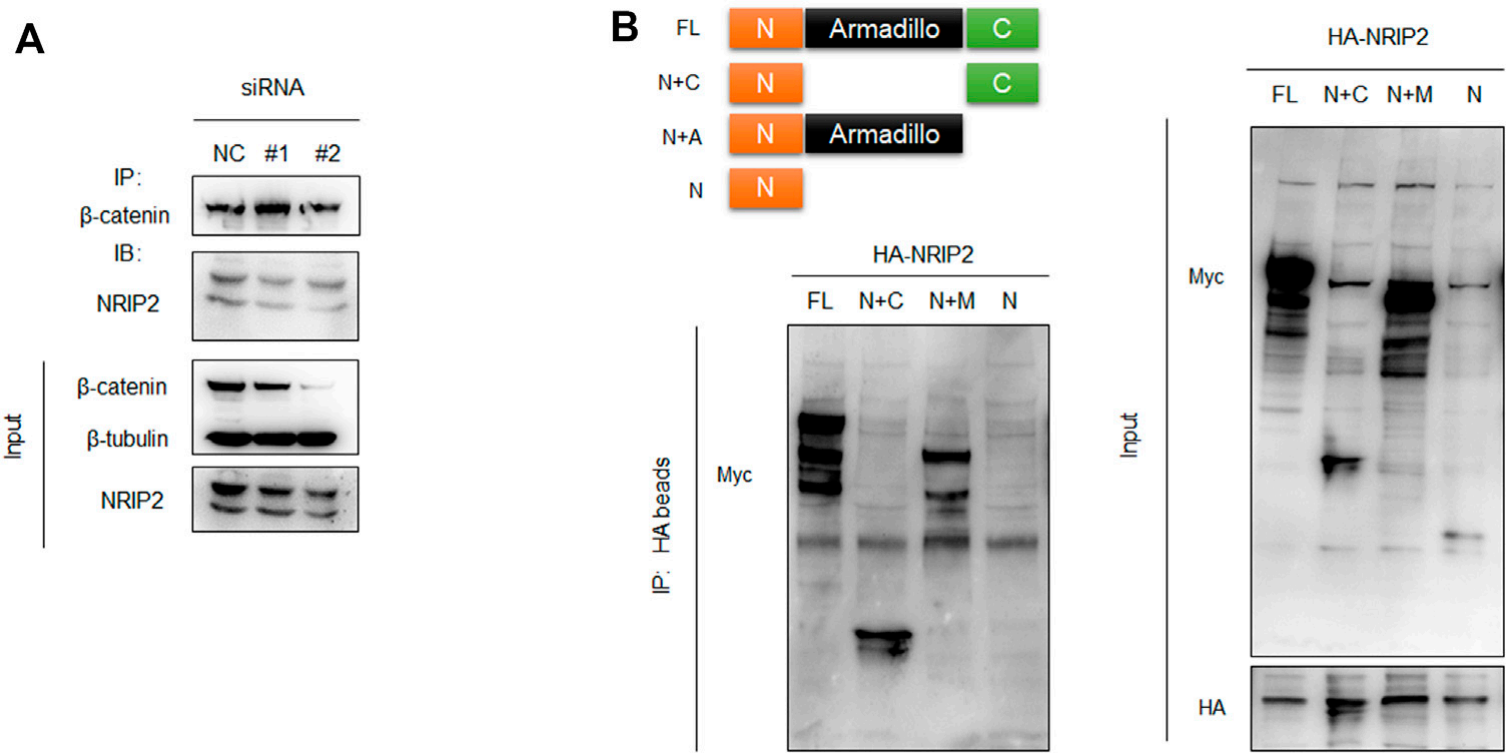
NRIP2 binds to β-catenin in podocytes. **(A)** Coimmunoprecipitation (co-IP) of endogenous NRIP2 with β-catenin in podocytes. β-Catenin was immunoprecipitated, and the amount of NRIP2 bound to β-catenin was determined using immunoblotting analysis. Cells transfected with siRNAs targeting NRIP2 were used as negative controls. **(B)** The N-terminal domain of β-catenin mediates the interaction of β-catenin with NRIP2. Schematic illustration of full-length β-catenin and β-catenin mutants. Myc-tagged β-catenin and its deletion mutants lacking the C-terminus, N-terminus and armadillo domain were co-transfected with HA-NRIP2 in podocytes. After 24 h of transfection, the cells were lysed and subjected to IP with HA beads followed by IB with anti-Myc and anti-HA antibodies. FL, β-catenin full length; N+C, β-catenin full length lacking Armadillo domain; N+A, β-catenin full length lacking C terminus; N, β-catenin full length lacking both Armadillo domain and C terminus.

To investigate a direct role for NRIP2 in regulating β-catenin, we first examined whether NRIP2 and β-catenin are expressed under physiological conditions. We used whole-cell extracts for an immunoprecipitation assay, and the interaction between endogenous NRIP2 and β-catenin was detected in cultured human podocytes (Figure 1A). Furthermore, to delineate the region in β-catenin responsible for its interaction with NRIP2, we co-transfected different β-catenin deletion mutants as indicated in (Figure 1B) with NRIP2, and immunoprecipitation showed that deletion of the N-terminus changed the binding affinity of β-catenin for NRIP2; thus, the N-terminal domain of β-catenin mediates the interaction between NRIP2 and β-catenin.

### NRIP2 is Required for β-Catenin Stabilization

**FIGURE 2:**
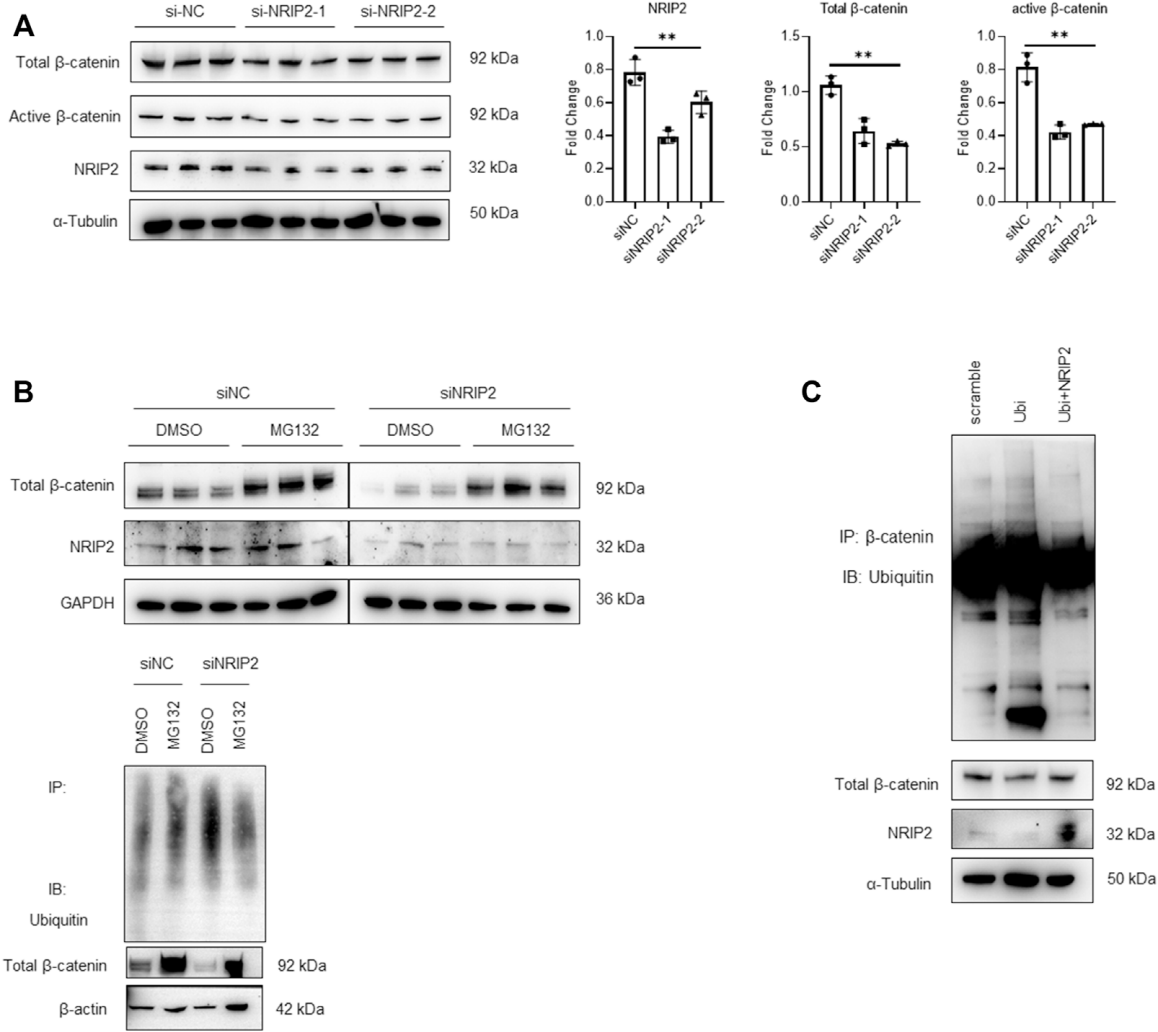
NRIP2 stabilizes β-catenin in podocytes. **(A)** Representative western blots showing quantification of NRIP2, total β-catenin and active β-catenin protein levels in podocytes transfected with negative control siRNA (siNC) or human NRIP2 siRNA (siNRIP2). **(B)** Representative western blots of total β-catenin protein levels in si-NC- and si-NRIP2-transfected podocytes incubated with 5 µM MG132 for 1 h, and immunoprecipitated with an anti-β-catenin antibody to detect the amount of ubiquitin bound to β-catenin. **(C)** Podocytes were transfected with the indicated plasmids. Cells were lysed and immunoprecipitated with an anti-β-catenin antibody, and the amount of ubiquitin bound to β-catenin was determined using immunoblot analysis.

**FIGURE 3:**
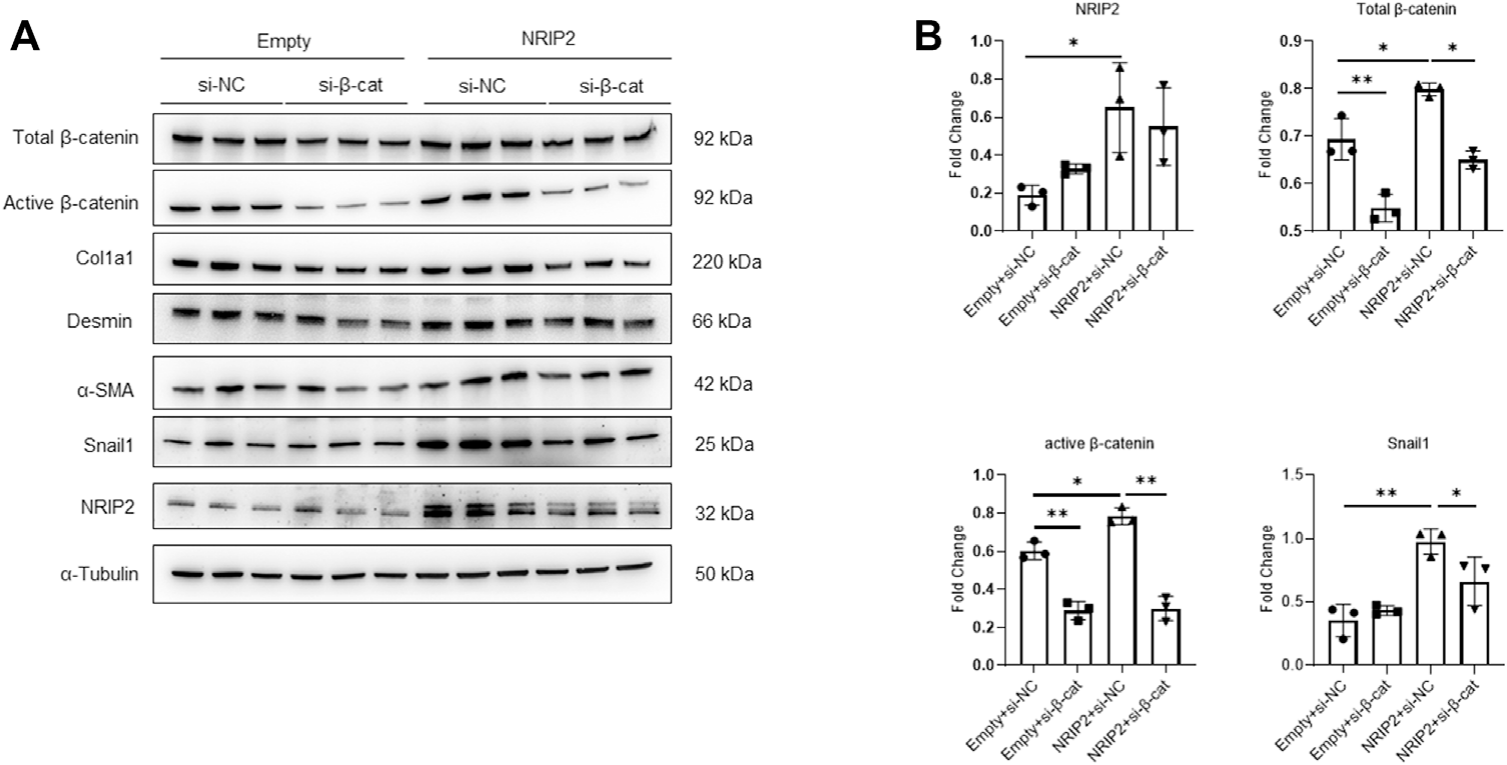
Overexpression of NRIP2 activates β-catenin. **(A,B)** Representative western blots with quantification of the protein levels of NRIP2, total β-catenin, active β-catenin and downstream target proteins in normal control and NRIP2-overexpressing podocytes transfected with si-NC or si-β-catenin as indicated. Statistical analysis was performed with two-tailed unpaired Student’s t test. *p < 0.05, **p < 0.01.

Subsequently, we examined whether NRIP2 regulates the abundance of β-catenin. NRIP2 knockdown with siRNA (#2 siRNA was used in the following experiments) decreased the levels of both total and active β-catenin (Figure 2A); conversely, MG132 treatment successfully restored the total β-catenin levels, suggesting that NRIP2 knockdown enhances β-catenin degradation by the proteasome (Figure 2B). To confirm the role of NRIP2 in preventing β-catenin degradation, we performed a ubiquitination assay. Comparing with control, NRIP2 knockdown enhanced β-catenin ubiquitination but reversed by MG132 (Figure 2B), in line with this, transient transfection of HA-tagged ubiquitin in podocytes resulted in increased levels of β-catenin ubiquitination; conversely, transfection of NRIP2 or treatment with MG132 blocked β-catenin ubiquitination (Figure 2C). In addition, transient overexpression of NRIP2 led to potent activation of β-catenin and induction of α-SMA, desmin and Col1a1, and these effects were attenuated by silencing β-catenin (Figures 3A,B). These results suggest that NRIP2 is critical for β-catenin stabilization and activation through the ubiquitin proteasomal pathway.

### NRIP2 is Critical for ADR-Induced β-Catenin Activation *in vitro* and *in vivo*

**FIGURE 4:**
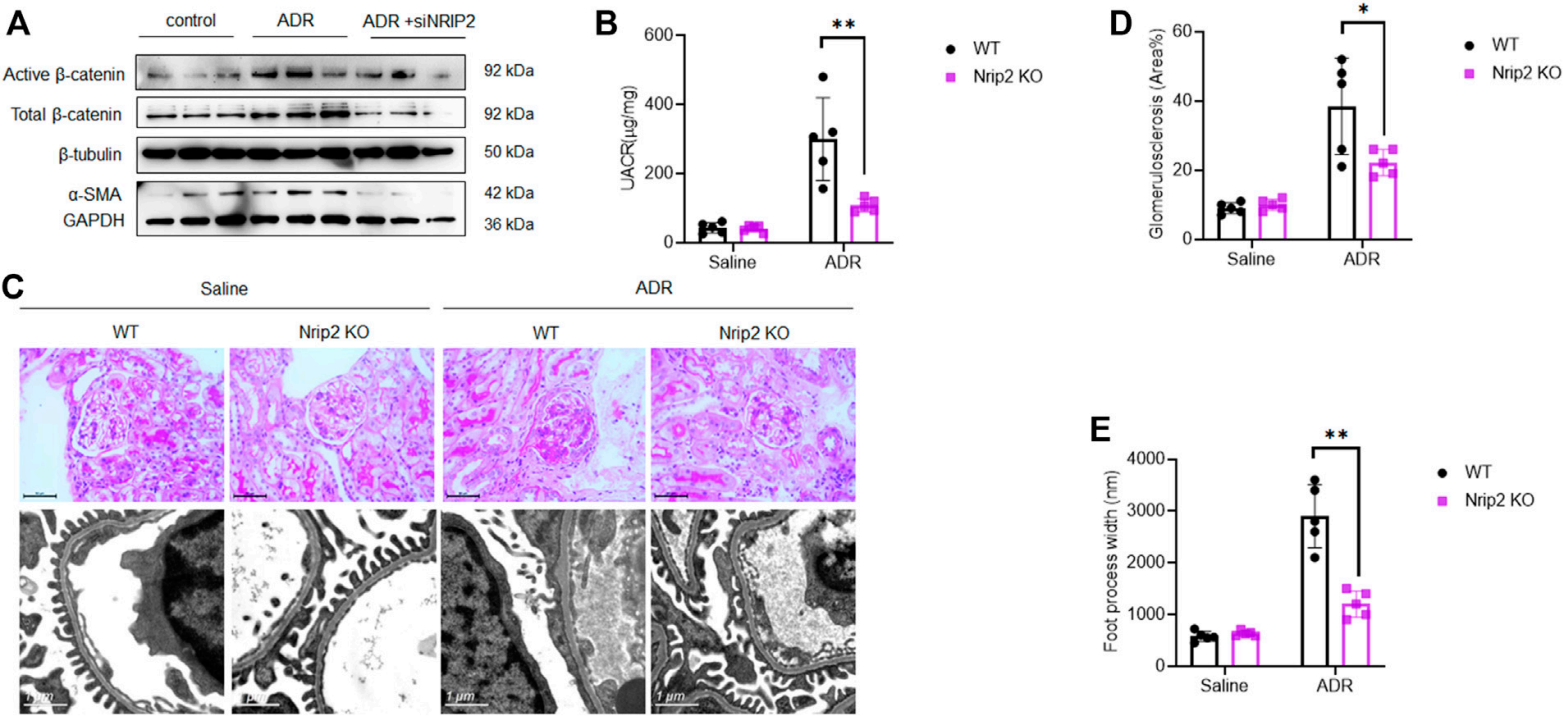
Loss of Nrip2 reduced ADR-induced podocyte injury and proteinuria *in vitro* and *in vivo*. **(A)** Representative western blots of total β-catenin, active β-catenin, NRIP2 and α-SMA in ADR-treated podocytes with or without si-NRIP2 transfection. **(B)** Urinary albumin/creatinine ratio (µg/mg) in the control WT, control KO, ADR-WT, and ADR-KO groups. **(C)** Representative PAS staining and TEM images in the control WT, control KO, ADR-WT, and ADR-KO groups. **(D)** Quantification of glomerulosclerosis in the four groups. **(E)** Quantification of foot process width (nm) in the four groups. *N* = 5 in each group; the data are shown as the mean ± SD values; statistical analysis was performed with two-tailed unpaired Student’s t test. *p < 0.05, **p < 0.01. ***p < 0.001.

β-Catenin signalling plays a central role in mediating podocyte dedifferentiation and mesenchymal transition. We sought to ascertain the role of NRIP2 in regulating β-catenin signalling during podocyte injury and found that NRIP2 knockdown greatly inhibited ADR-induced expression of total and active β-catenin (Figure 4A), translocation of β-catenin into nucleus were also confirmed by IF staining (Supplementary Figure S2).

To investigate the protective effects of NRIP2 deletion in a murine model of experimental nephropathy, we established a model of ADR nephropathy. We generated mice with global allelic knockout of Nrip2 using the CRISPR/Cas9 approach (Supplementary Figure S1A) on a C57BL/6N background, which exhibits ADR susceptibility (Arif et al., 2016). Loss of Nrip2 in the knockout (KO) mice was confirmed by western blotting (WB) of kidney cortices (Supplementary Figure S1B). No developmental defects were observed in Nrip2 KO mice.

**FIGURE 5:**
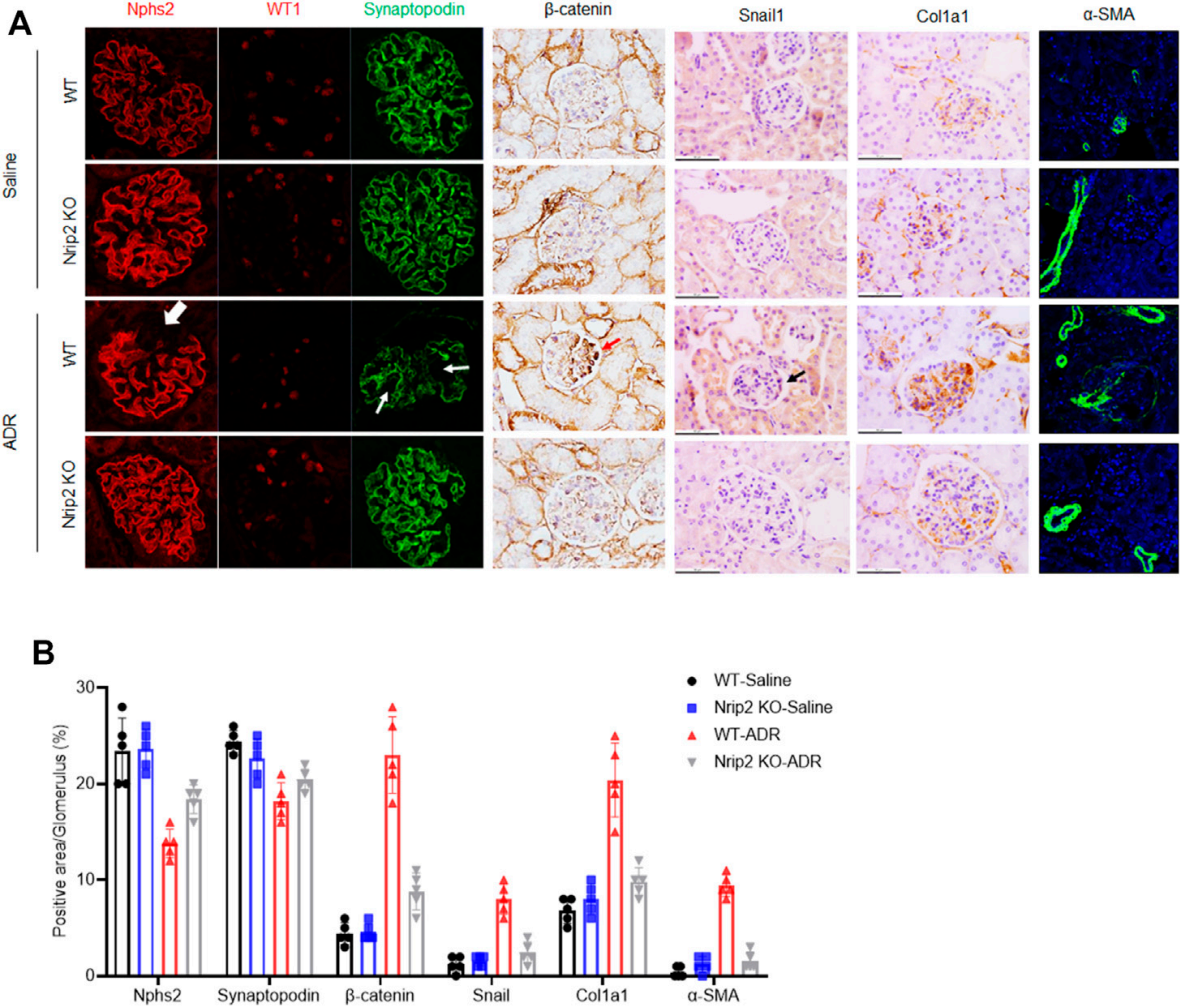
NRIP2 is required for β-catenin activation during podocyte injury. **(A)** Immunohistochemical (IHC) and immunofluorescence (IF) staining showed the expression patterns of β-catenin and its downstream target genes as well as podocyte markers, including synaptopodin, Nphs2 and WT1, in different groups as indicated. **(B)** Representative quantification in the glomerulus. Statistical analysis was performed with two-tailed unpaired Student’s t test. *p < 0.05. *p < 0.01.

As expected, ADR-treated WT mice developed severe albuminuria, while ADR-treated KO mice displayed much lower proteinuria levels (Figure 4B). Histological examination using light microscopy and transmission electron microscopy (TEM) (Figure 4C) revealed that ADR-treated WT mice developed segmental glomerulosclerosis (Figure 4D) and podocyte foot process effacement (Figure 4E), while ADR-treated KO mice displayed a normal histological structure. Immunostaining (Figures 5A,B) confirmed the downregulation of podocin, synaptopodin and WT1 and the upregulation of β-catenin, snail1, α-SMA and Col1a1 in ADR-treated WT mice. Comparatively, the expression of podocin, synaptopodin and WT1 was normal in ADR-treated KO mice, and the activation of β-catenin and its target genes snail1, α-SMA and Col1a1 was blocked in ADR-treated KO mice. These data demonstrate that loss of Nrip2 ameliorates podocyte injury, proteinuria and glomerulosclerosis by inhibiting β-catenin activation.

### NRIP2 is Upregulated in Podocytes From NS Patients

**FIGURE 6:**
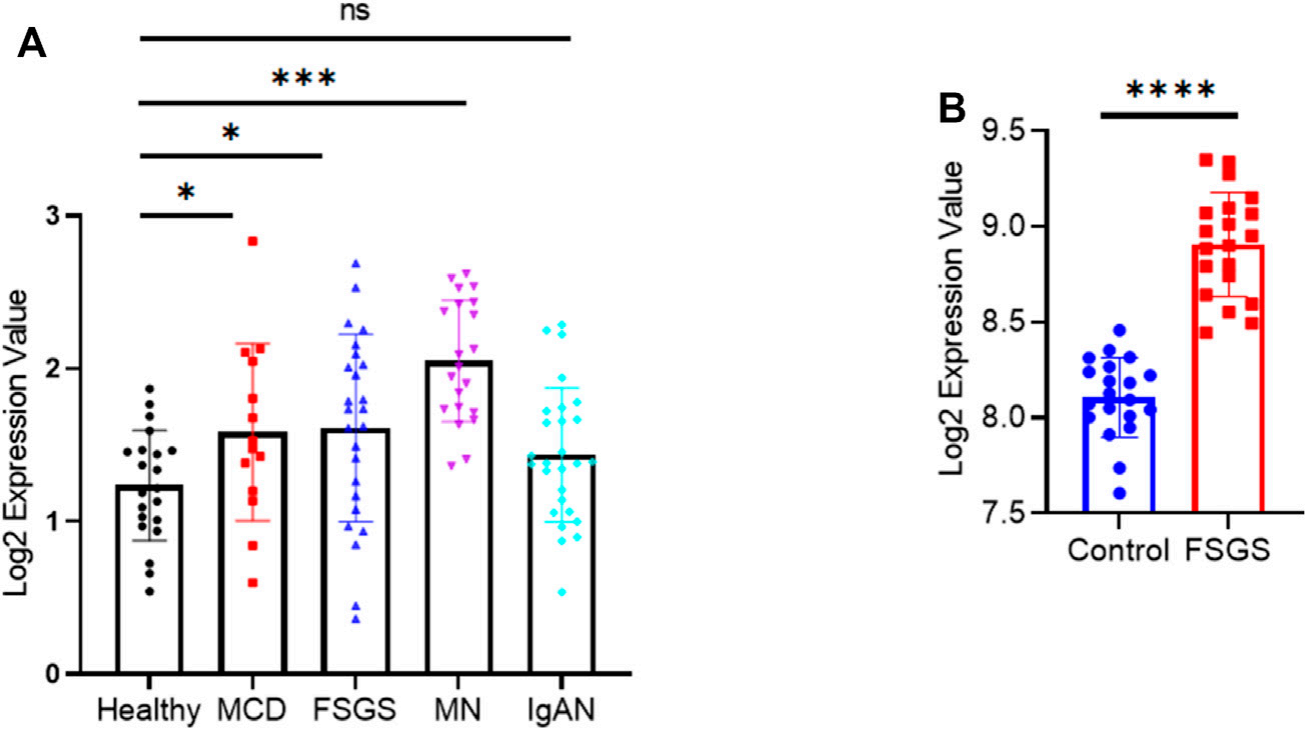
NRIP2 expression in human kidneys. **(A)** NRIP2 mRNA expression in dissected glomeruli from healthy living donor and NS kidney biopsy samples (Ju CKD). Statistical analysis was performed with a 2-tailed, unpaired t test. **(B)** NRIP2 mRNA expression in dissected glomeruli from healthy living donor and FSGS kidney biopsy samples, *N* = 20. statistical analysis was performed with two-tailed unpaired Student’s t test. *p < 0.05, **p < 0.01, ***p < 0.001, ****p < 0.0001.

**FIGURE 7:**
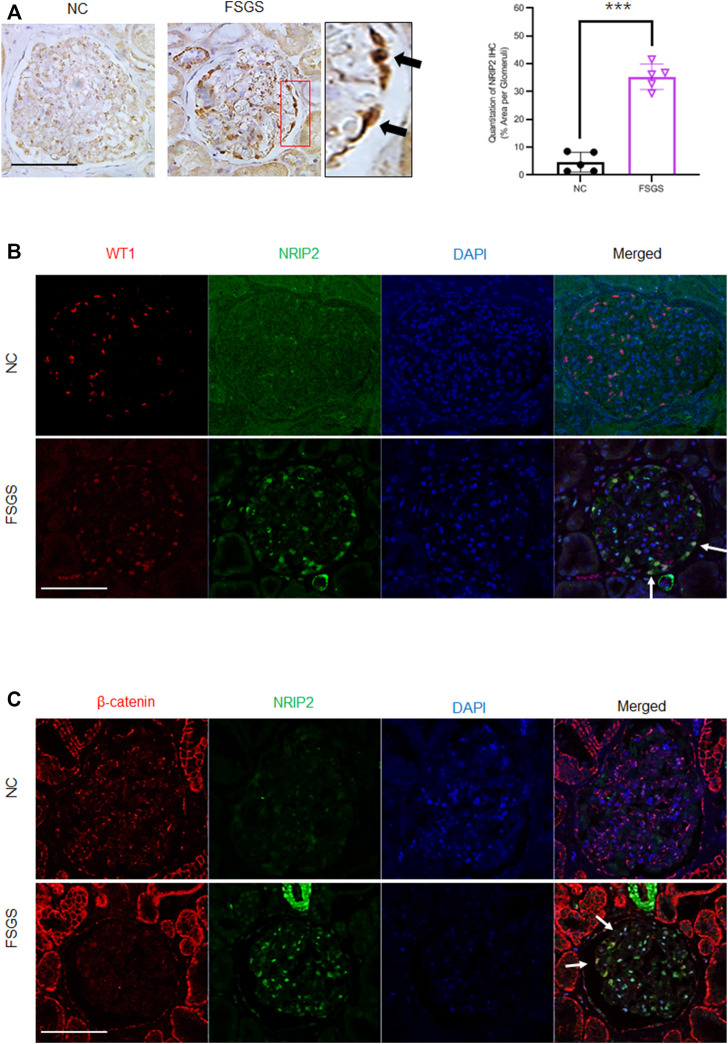
Validation of NRIP2 expression in human kidney samples. **(A)** Representative IHC staining of NRIP2 (black arrows) and quantification in the glomerulus. *N* = 5; statistical analysis was performed with a 2-tailed, unpaired t test. **(B)** IF staining image clearly showing NRIP2 localized with WT1 (white arrows) in podocytes from healthy control and human patients with FSGS; scale bar: 50 µm. **(C)** Representative images of IF staining clearly showing NRIP2 localized with β-catenin (white arrows) in podocytes from human patients with FSGS but not in control podocytes; scale bar: 50 μm statistical analysis was performed with two-tailed unpaired Student’s t test. ***p < 0.01.

To determine whether our findings have clinical relevance, we examined NRIP2 expression in human kidneys using the Nephroseq database (http://www.nephroseq.org) (Ju et al., 2015), which showed (Figure 6A) that glomerular expression of NRIP2 was induced in kidneys from human patients with minimal change disease (MCD), FSGS and membranous nephropathy (MN) but not in kidneys from those with IgA nephropathy (IgAN). We also examined NRIP2 expression using an internal microarray dataset (GEO: GSE129973), which showed (Figure 6B) that glomerular expression of NRIP2 was also upregulated in kidneys of human patients with FSGS. We further determined that the glomerular NRIP2 level was increased in biopsy samples of FSGS patients in comparison with normal controls. IHC staining (Figure 7A) of NRIP2 in biopsy samples of FSGS patients confirmed its predominant expression in podocytes, where it was colocalized with WT1 (Figure 7B). We also observed the colocalization of NRIP2 and β-catenin in biopsy samples of FSGS patients by immunofluorescence staining (Figure 7C). These results therefore confirmed a role of NRIP2 in podocyte injury.

## Discussion

In summary, the present work revealed a role of NRIP2 in stabilizing β-catenin in podocytes, and this role of NRIP2 is involved in podocyte injury in NS patients.

β-Catenin signalling has a central role in mediating podocyte damage and proteinuria. During podocyte injury, β-catenin activation is dependent on receptor of advanced glycation end products (RAGE)-mediated NADPH oxidase induction, reactive oxygen species generation, and nuclear factor-κB activation (Zhou et al., 2019); β-catenin activation is negatively regulated by peroxisome proliferator-activated receptor γ (PPARγ) (Zhou et al., 2017) and signal regulatory protein α (SIRPα) (Li et al., 2019). β-Catenin is also regulated by miR-466o-3p (Chen et al., 2020) and the lncRNA MALAT1 (Hu et al., 2017), and protein kinase C stabilizes β-catenin and regulates its subcellular localization in podocytes (Duong et al., 2017). However, the regulation of β-catenin in podocytes is still largely uncharacterized.

Stabilization of β-catenin is important for its nuclear translocation and subsequent activation (Yang et al., 2011; Griffin et al., 2018). To date, a number of β-catenin stabilizers have been identified. For example, DHX32 interacts with and stabilizes β-catenin to promote angiogenesis in colorectal cancer cells (Lin et al., 2017), the redox-sensitive enzyme SENP3 interacts with β-catenin and inhibits its proteasome-dependent degradation in vascular smooth muscle cells (Cai et al., 2021), and MRP4 sustains β-catenin signalling by binding to β-catenin and blocking its degradation in the receptive endometrium to facilitate IVF (Chen et al., 2019).

Here, we describe that NRIP2 is necessary for β-catenin stabilization and activation in podocytes. Enhanced β-catenin degradation was observed in NRIP2-knockdown podocytes but was blocked by MG132 treatment, which means that β-catenin degradation is ubiquitin–proteasome-dependent. The interaction of NRIP2 and β-catenin was required for β-catenin stabilization. Additionally, reinforced expression of NRIP2 in podocytes was sufficient to activate β-catenin. Collectively, our findings indicate that NRIP2 interacts with and stabilizes β-catenin in podocytes.

Furthermore, our *in vitro* and *in vivo* data demonstrate that NRIP2 is required for β-catenin activation during podocyte injury. *In vitro*, β-catenin activation was dramatically activated in ADR-treated podocytes but totally blocked in NRIP2-knockdown podocytes. *In vivo*, NRIP2 knockout ameliorated podocyte injury and proteinuria in mice with ADR nephropathy by inhibiting β-catenin activation and the expression of its downstream target genes. In NS patients, glomerular expression of NRIP2 was found to be significantly upregulated and mainly localized in podocytes. The interaction of NRIP2 and β-catenin was observed in podocytes from FSGS patients. These data summarize the importance of NRIP2 for β-catenin activation during podocyte injury.

It is well known that β-catenin signalling is essential for kidney development (Park et al., 2007; Boivin et al., 2015; Drake et al., 2020), but no kidney developmental defects are observed in NRIP2 KO mice. The limitations of this study are as follows: 1) we could not preclude the effects of NRIP2 loss in cells other than podocytes, 2) we did not explore which E3 ubiquitin ligase competes with NRIP2 to recognize β-catenin in podocytes, and 3) whether NRIP2 cooperates with β-catenin to drive target gene expression remains unknown, because NRIP1 (Vivante et al., 2017) encodes a nuclear receptor transcriptional cofactor that directly interacts with RARs to modulate retinoic acid transcriptional activity during kidney development.

In conclusion, we describe a previously unsuspected protein–protein interaction between NRIP2 and β-catenin and show that this interaction is important for stabilizing β-catenin in podocytes.

## Data Availability

All relevant data are available from the authors on request and/or are included with the article (as figure source data or Supplementary Material). The accession number for the microarray raw data reported in this work is GEO: GSE129973.
