# Supplementary material for: Nuclear Receptor Interacting Protein-2 Mediates the Stabilization and Activation of β-Catenin During Podocyte Injury
Source: Front Cell Dev Biol. 2021 Dec 24;9:781792. doi: 10.3389/fcell.2021.781792 (PMC8740220; doi:10.3389/fcell.2021.781792)
Supplement: Supplementary file 1 [file DataSheet1.pdf]

Supplementary Table 1. baseline characteristics at the time of renal biopsy

	Control	FSGS
Cases	N= 20	N= 20
Age	36.0 (30.0 – 39.3)	28.3 (23.0 – 40.8)
Female (%)	40% (8)	40% (8)
Proteinuria (g/24h)	-	6.6 (3.4 – 9.3)
ALB (g/L)	NA	22.0 (20.2 – 24.6)
eGFR	NA	83.4 (65.1 – 111.8)

Supplementary Figure 1. Generation strategy of NRIP2 KO mice

A

B

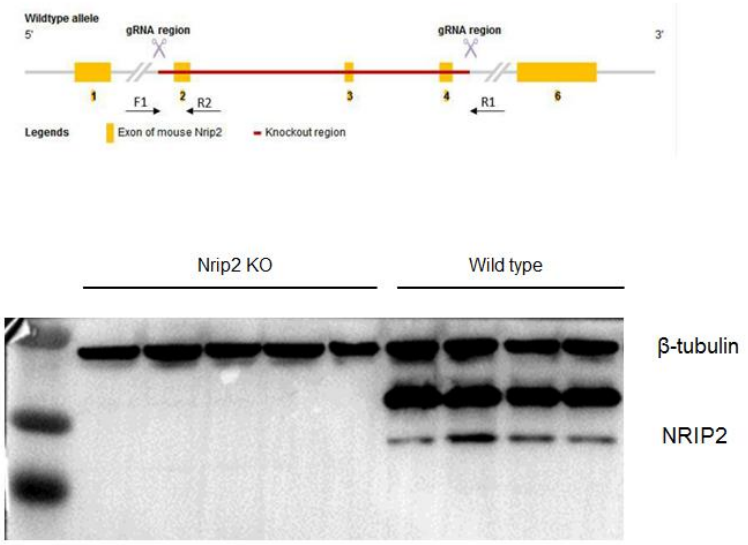

Supplementary Figure 2. β-catenin IF staining in cultured human podocytes treated with or without ADR

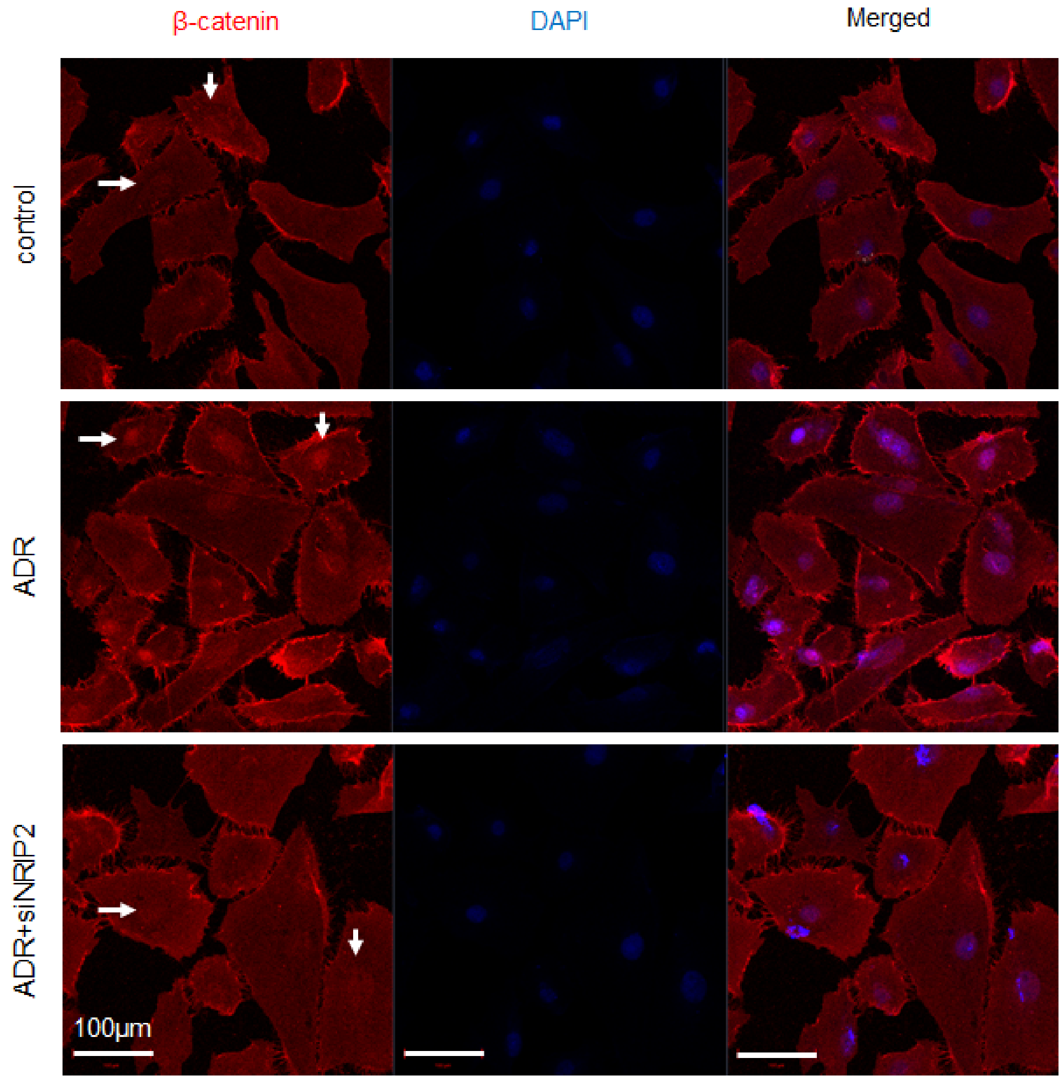
β-catenin
DAPI
Merged
control
ADR
ADR+siNRIP2
100μm
